# Limited Access to Blinatumomab in Morocco: Challenges and Implications

**DOI:** 10.7759/cureus.110511

**Published:** 2026-06-09

**Authors:** Nabiha El Abdi, Yasmine El Ouafa, Fatima Azzahra Lahlou, Nouama Bouanani

**Affiliations:** 1 Interdisciplinary Laboratory of Biothechnology and Health, Mohammed VI University of Health Sciences (UM6SS), Casablanca, MAR; 2 Center for Doctoral Studies (CEDoc), Mohammed VI University of Health Sciences (UM6SS), Casablanca, MAR; 3 Biochemistry, Faculty of Medicine, Mohammed VI University of Health Sciences (UM6SS), Casablanca, MAR; 4 Hematology, Faculty of Medicine, Mohammed VI University of Health Sciences (UM6SS), Casablanca, MAR

**Keywords:** acute lymphoblastic leukemia, b-cell acute lymphoblastic leukemia, bispecific antibodies, blinatumomab, hematology, morocco

## Abstract

Recent advances in hematology, particularly immunotherapy, have significantly improved outcomes in acute lymphoblastic leukemia (ALL). Blinatumomab, a CD19-directed bispecific T-cell engager, has demonstrated substantial efficacy in relapsed or refractory B-cell ALL, achieving high rates of minimal residual disease negativity and improving overall survival.

However, there are still disparities in how these therapeutic advancements are incorporated into standard clinical practice across the globe. Financial obstacles, restricted drug availability, and regulatory delays often impede access to novel agents like blinatumomab in Morocco and other middle-income countries. As a result, despite the mounting evidence in favor of immunotherapy-driven approaches, many patients still receive traditional chemotherapy.

This editorial highlights the gap between international standards of care and real-world practice in Morocco and underscores the urgent need to improve equitable access to life-saving hematologic therapies.

## Editorial

Therapeutic advances in hematology have significantly improved the prognosis for malignant blood disorders. In adults, particularly in acute lymphoblastic leukemia (ALL), the development of immunotherapies such as blinatumomab, a bispecific antibody that plays a key role in activating T cells targeting CD19, has revolutionized treatment strategies, especially in cases of relapse or recurrence [1,2]. Despite these advances, access to innovative treatments remains limited in many middle-income countries, including Morocco, leading to a significant gap between international guidelines and actual clinical practice.

Blinatumomab, a bispecific antibody targeting CD19 and CD3, has significantly changed the management of B-cell ALL in adults with relapsed or refractory disease. Clinical trials, including the TOWER and BLAST studies, have demonstrated that blinatumomab significantly improves overall survival and achieves high rates of measurable residual disease (MRD) negativity, a strong predictor of durable remission [1,2].

Epidemiological data from Morocco indicate that leukemia constitutes a significant proportion of hematologic malignancies. A retrospective study in Eastern Morocco reported the distribution and clinical features of hematologic cancers over five years, confirming the predominance of acute leukemias among adults [3]. Similarly, data from the Casablanca Cancer Registry, covering approximately 3.6 million inhabitants, show that hematopoietic and lymphoid malignancies account for 11-13% of all cancers, with leukemia representing a substantial fraction and age-standardized incidence rates of 2.7 per 100,000 men and 2.0 per 100,000 women [4]. Overall, these findings highlight that adult acute leukemias represent a significant burden in Morocco, despite the limited availability of comprehensive national data.

Although blinatumomab has yielded remarkable results internationally, its practical use in the treatment of ALL in Morocco remains limited. Limited access to this targeted therapy, due to high costs, regulatory delays, and restricted reimbursement, continues to restrict its use, leaving many patients reliant on conventional chemotherapy rather than evidence-based, more effective options. Regulatory and logistical hurdles continue to hinder access to blinatumomab in Morocco. Even after international approval, high costs, slow reimbursement processes, and limited local availability frequently delay or prevent patients from receiving this standard-of-care therapy in a timely manner [5].

In resource-limited countries, access to blinatumomab starkly illustrates global inequalities in hematology. In addition to its high cost, this treatment poses specific organizational challenges, particularly the administration of blinatumomab via continuous infusion, which requires appropriate infrastructure and trained staff. In this context, strategies have been proposed to improve feasibility in LMICs, including shortened treatment regimens, vial sharing, and outpatient administration to reduce costs while maintaining clinical efficacy [6]. These approaches underscore that access to this immunotherapy depends more on organizational innovations than on therapeutic advances themselves.

In addition, challenges related to the implementation of blinatumomab in low- and middle-income countries (LMICs) have been identified in the literature as well, including financial, logistical, and healthcare infrastructure barriers. These barriers may play a role in limited and inequitable access across such settings. Blinatumomab has demonstrated robust efficacy in international clinical trials, but is not widely available in these countries in real-world practice [7].

In LMICs such as Mexico, access to targeted therapies is frequently limited by financial constraints, insufficient infrastructure, and disparities in healthcare resources. As a result, treatment protocols originally designed for high-income countries are often difficult to implement in routine clinical practice. These realities highlight the importance of developing pragmatic and context-adapted strategies to expand patient access to innovative therapies. Adjustments in dosing, treatment duration, or administration schedules may represent practical and evidence-based alternatives to improve feasibility, optimize resource utilization, and promote more equitable cancer care [8].

A descriptive cost analysis based on strategies for sharing vials and optimizing doses was conducted, and the 12 patients were treated using 75 vials of blinatumomab instead of 132 vials [6].

To address these challenges, it is important to implement a range of strategies at various levels. Reducing approval times at the national level, facilitating the integration of innovative hematological treatments such as blinatumomab into clinical practice, and promoting value-based treatment decisions are essential steps toward improving and strengthening equity in care.

In a context where immunotherapy in hematology plays a very important role in the management of ALL, unequal access to innovative therapies in resource-limited countries and economic and regulatory constraints may amplify inequalities in care and the impact on patient survival. Hence, there is a need to ensure the rapid and affordable availability of these treatments through national cancer control policies.

Finally, it will be essential to evaluate the impact of limited access to innovative treatments, such as blinatumomab, on survival and quality of life in additional studies to guide future health strategies.
